# Biochemical outcome after curative treatment for localized prostate cancer with external beam radiotherapy: a cross-sectional study

**DOI:** 10.3332/ecancer.2023.1625

**Published:** 2023-11-10

**Authors:** Joseph Daniels, Kofi Adesi Kyei, Kikelomo Adeola Badejoko–Okunade, Samuel Anim-Sampong, Samuel Nii Adu Tagoe, William Kwadwo Antwi, Joana Ainuson-Quampah, Adewumi Alabi, Anthonia Sowunmi, Judith Naa Odey Tackie

**Affiliations:** 1 National Centre for Radiotherapy, Oncology and Nuclear Medicine, Korle Bu Teaching Hospital, PO Box KB 369, Korle Bu, Accra, Ghana; 2 Department of Radiography, University of Ghana, Legon, PO Box KB 143, Korle Bu, Accra, Ghana; 3 Lagos University Teaching Hospital, Ishaga Road, PO Box 102215, Lagos, Nigeria; 4 Department of Dietetics, University of Ghana, Legon, PO Box KB 143, Korle Bu, Accra, Ghana

**Keywords:** androgen deprivation therapy, biochemical recurrence, localised prostate cancer, external beam radiotherapy, time to biochemical progression, prostate-specific antigen, prostate adenocarcinoma, radiotherapy, intensity-modulated radiotherapy

## Abstract

Although many patients who receive definitive radiotherapy (RT) for localised prostate cancer (CaP) experience long-term disease-free survival and better quality of life, some also have biochemical progression during follow-up. Oftentimes this implies additional treatment for patients with the accompanying challenges of cumulative treatment side effects, inconvenience and financial toxicity. This study retrospectively assessed the clinicopathological characteristics and biochemical outcomes of patients treated for localised CaP with external beam radiotherapy (EBRT) between 2015 and 2020 at a major cancer treatment centre in Accra, Ghana. Patients’ socio-demographic and clinical data were collected from their hospital records and analysed with the Statistical Package for Social Sciences version 26. Biochemical failure (BCF) was defined as an increase in the level of serum prostate-specific antigen (PSA) >2 ng/mL above the nadir after curative therapy based on the Phoenix definition. The mean age was 67.6 years (SD ± 6.2). The majority of the study participants (*n* = 79, 64.8%) had initial PSA >20 ng/mL, with the highest recorded value of 705 ng/mL. All the patients had biopsy-proven adenocarcinoma of the prostate gland. Some patients received 3-dimensional conformal radiotherapy (3DCRT) on a cobalt-60 teletherapy machine whereas others were treated with either 3DCRT or intensity-modulated radiotherapy (IMRT) on a 6 MV Linac. In all, 13.1% of the patients experienced BCF after receiving EBRT after an average follow-up of 31.3 months. This study demonstrated a low rate of BCF among patients treated with EBRT for localised CaP in Ghana. Strong prognostic factors of biochemical outcome demonstrated in this study were the percentage of cores positive, grade group, and risk stratification. Diarrhaea and desquamation experienced by treated CaP patients were exclusively attributable to EBRT. RT produced a complete resolution of symptoms in some of the patients.

## Introduction

Prostate cancer (CaP) is a significant public health problem around the globe and a prime cause of morbidity and mortality among adult men. CaP was the second most frequently diagnosed cancer among men and the fifth leading cause of cancer deaths among males in 2020 [1] . The incidence of CaP is highest in North America, Northern and Western Europe, as well as Oceania. It is also predominant among black men in the United States of America. It is also reported that males of African descent are at an increased risk of developing CaP [2]. CaP is the most frequently diagnosed cancer and primary cause of cancer mortality among Ghanaian men [3]. A median incidence rate of 19.5 per 100,000 population has been reported across the African continent [4]. East African countries have the highest incidence rates ranging from 10.7 to 38.1 per 100,000 men, whereas West African countries have the lowest incidence rates ranging from 4.7 to 19.8 per 100,000 men [5]. In Ghana, the reported incidence rate is 10.5 per 100,000 men [2, 4].

Based on the local extent of spread of CaP, involvement of regional lymph nodes and distant metastatic sites, the disease can be divided into three groups: localised CaP (T1, T2 and early T3N0M0), locally advanced CaP (T3 or T4, Any N, M0 or Any T, N1, M0) and metastatic CaP (Any T, Any N, M1). Serum prostate-specific antigen (PSA) is the most widely used tumour marker in CaP screening and surveillance after treatment. It is by far, the most accurate indicator of CaP risk [6]. Serum PSA has a normal upper limit of 4.0 ng/mL. High serum PSA levels correlate with a high probability of positive tissue diagnosis, increased risk of bone metastases and high Gleason score [7].

Based on the highest pretreatment PSA value, digital rectal examination findings and the Gleason score from prostate biopsy, CaP can be stratified into different risk groups that determine the standard of care for each patient. These are the very high, high, unfavourable intermediate, favourable intermediate, low and very low risk groups. The high-risk group is characterised by extra prostatic disease, and/or PSA >20 ng/mL and/or Gleason score 8 to 10. The intermediate risk group has cancer limited to the prostate with PSA between 10 and 20 ng/ml and a Gleason score of 7. On the other hand, the low-risk groups (both low and very low risk) are characterised by tumours that are confined to the prostate gland with PSA <10 ng/mL and Gleason score of 6.

Patients with low-risk diseases have the option of receiving either immediate active treatment, active surveillance or observation. Active treatment can be delivered as external beam radiotherapy (EBRT), brachytherapy or radical prostatectomy. Patients with intermediate risk CaP can choose therapy with EBRT or brachytherapy in combination with hormonal therapy. A radical prostatectomy is also an option for this group of patients as primary therapy followed by adjuvant treatment depending on the surgical findings. Active surveillance and observation can also be considered on a case-by-case basis depending on the performance status, comorbidities, life expectance and aggressive nature of patient’s malignant disease. The high-risk groups require either radical prostatectomy or radiation treatment with hormonal (androgen deprivation) therapy (ADT) up to 3 years. In some instances, abiraterone or docetaxel (a chemotherapeutic agent) can be added to radiation treatment and ADT.

Radiotherapy (RT) has been found to increase the likelihood of freedom from biochemical failure (BCF), especially in low risk localised CaP [8]. RT plays a key role in the therapeutic management of CaP, with nearly 25% of patients younger than 65 years and 40% of patients aged ≥65 years receiving either EBRT or prostate brachytherapy [9]. Previous studies have shown that RT can significantly improve biochemical control in people with localised CaP thus justifying its use in the management of the disease [10]. Although many patients who receive definitive RT for localised CaP experience long disease-free survival and better quality of life, some also have biochemical progression during follow-up [11].

BCF refers to the persistence or recurrence of elevated serum PSA levels after completion of definitive curative therapy for CaP and is indicative of inadequately controlled or progressive disease. The exact criteria for defining BCF depends on the treatment modality and medical guidelines used. Typically, after radical prostatectomy, serum PSA levels are expected to drop to undetectable levels within 6 weeks after surgery. If PSA levels remain detectable or become detectable (>0.2 ng/mL) after a period of being undetectable, then there is BCF [12]. After treatment with EBRT or brachytherapy, BCF refers to either three consecutive rises in serial PSA >0.2 ng/mL or a rise in PSA >2 ng/mL above the nadir per the Phoenix criteria [9, 13]. BCF does not necessarily imply clinical failure. It is estimated that about 24%–34% of patients who develop BCF experience clinical failure within 15 years of surgery [14]. The appearance of clinical failure may occur up to 8 years after radical prostatectomy or 7 years after radiation therapy [15, 16].

BCF may require additional treatment for patients which is associated with increased toxicity, financial responsibilities and inconvenience. Despite the plethora of published literature on CaP, few studies have focused on the biochemical outcomes of patients who are treated with definitive radiation therapy. Currently. there is limited information about the prevalence and impact of BCF on patients treated for localised CaP in Ghana. Additionally, little is known about the clinicopathological characteristics these patients in the Ghanaian setting. This study was therefore conducted to retrospectively assess the clinicopathological characteristics and biochemical outcomes of patients treated for localised CaP with EBRT in Ghana.

## Methods

This research was a single-institution cross-sectional study that evaluated the clinicopathological characteristics and biochemical outcomes of patients with localised CaP who were treated with EBRT between 1st January 2015 and 31st December 2020. In all, 122 patients participated in the study based on a total population sampling of all patients with histologically confirmed localised CaP who were treated during the sampling period. Patients received treatment either with a Cobalt-60 teletherapy machine or a 6 MV Varian linear accelerator (Linac). Some of the patients in this study received treatment with a standard four-field box technique whereas others were treated with a six-field conformal RT technique. A conformal cerrobend block was used during treatment with the Cobalt-60 machine whereas multileaf collimation was employed in the Linac. Localised CaP was defined as clinical T_1_N_0_M_0_, T_2_N_0_M_0_ or early T_3_N_0_M_0_ [17] per the National Comprehensive Cancer Network clinical practice guidelines in oncology. The evaluation and diagnostic staging of the patients entailed bone scans, chest radiographs and in some instances, abdominopelvic computed tomography (CT) scans to exclude distant metastasis. All patients in this study with either high or very high-risk CaP received either 2 years of adjuvant ADT with a gonadotropin releasing hormone agonist or underwent bilateral orchiectomy. Patients with unfavourable intermediate risk disease received short course adjuvant ADT with 6 months of Goserelin or Leuprolide therapy. On the other hand, CaP patients with either low or favorable intermediate risk disease did not receive ADT at all. Relevant data were extracted from patients’ medical records including demographic information, medical history, results of histopathological analysis, Tumour – Node – Metastasis (TNM) stage, Gleason score, initial PSA (iPSA), risk stratification and details of RT prescription. Serial follow-up PSA values at 3 monthly intervals post-RT were also recorded. BCF was defined as an increase in serum PSA ≥2 ng/mL above the nadir based on the Phoenix definition (nadir + 2 µg/L) [9, 12, 13]. Data were statistically analysed with the Statistical Package for Social Sciences version 26. Graphical representations such as pie charts and bar graphs as well as descriptive statistics in the form of frequency distribution, percentages, mean, and SDs were used to present results. Linear regression was used to establish the significance of the relationship between dependent and independent variables. A confidence interval of 95% was used and *p*-value < 0.05 was considered statistically significant. Informed consent was obtained from the patients for their participation in the study. Ethical approval was obtained from the institutional review board prior to commencement of the study. Confidentiality of information was maintained during the study and the data collected was anonymised with the removal of all traces of patient identifying information.

## Results

### Baseline characteristics

The mean age was 67.6 years (SD ± 6.2). The oldest patient was 81 years whereas the youngest was 53 years. Peak incidence was among patients between 60 and 69 years (*n* = 57, 46.7%). The majority of the patients (*n* = 115, 94.3%) had an Eastern Cooperative Oncology Group (ECOG) performance status of zero (0) at presentation whereas the remainder (*n* = 7, 5.7%) were ECOG 1 (Table 1). Fifty of the patients (41.0%) with localised CaP had a history of alcohol intake whereas 14 (11.5%) were smokers. Alcohol use and smoking histories were not specified for 20 (16.4%) of the patients. The majority of the patients had known comorbidities such as hypertensive heart disease, diabetes mellitus (DM) and ischemic heart disease. All the patients (100%) had adenocarcinoma of the prostate gland. In this study, 49 patients (40.2%) had <50% cores positive from biopsy samples whereas 61 patients (50%) had ≥50% cores positive. The percentages of cores positive were not specified for 12 patients (9.8%). The American Joint Committee on Cancer ‘TNM’ staging was used for classifying all patients. The study participants had varying T-categories but were all N_0_M_0_. T_2c_ was the commonest T category diagnosed among the patients (*n* = 45, 36.9%) whereas and the least common was T1a (*n* = 3, 2.5%). Patients were stratified into International Society for Urological Pathology (ISUP) grade groups 1 – 5 depending on their primary and secondary Gleason scores. The modal ISUP grade group was group 1 (*N* = 40, 32.8%) whereas the least frequent grade group was group 5 (*N* = 6, 5%). All the patients received EBRT with a conventional dose fractionation of 2 Gy per fraction per day for 5 days every week. Majority of the patients (*n* = 81, 66.4%) received a total dose of 74 Gy whereas 6.6% and 9.8% were treated with escalated doses of 76 Gy and 78 Gy, respectively. In all, 81 patients (66.4%) received 3-dimensional conformal radiotherapy (3DCRT) on the cobalt-60 treatment machine whereas (*n* = 41, 33.6%) were treated with either 3DCRT or intensity-modulated radiotherapy (IMRT) on the 6 MV Linac.

ISUP = International Society of Urological Pathology, ECOG = Eastern Cooperative Oncology Group, MV = Mega-voltage. All the patients treated to ≤72 Gy as well as 60 of the patients treated to a total radiation dose of 74 Gy were treated on the cobalt-60 teletherapy machine. On the other hand, all the patients treated to total doses of either 76 Gy or 78 Gy as well as 21 of the patients who received 74 Gy were treated on the 6 MV Linac. All patients treated on the cobalt-60 teletherapy machine were treated with 3-dimensional conformal radiotherapy whereas patients were treated with either 3-dimensional conformal radiotherapy or IMRT.

### Metastatic workup

**Figure 1.**
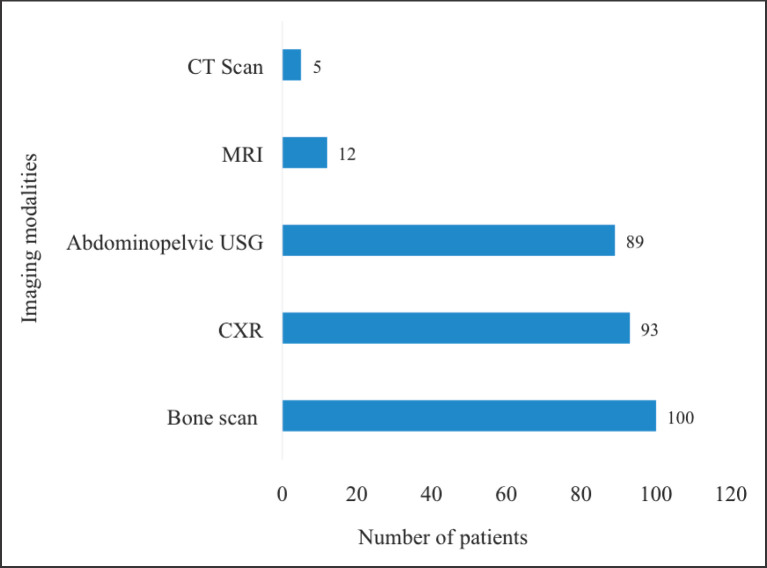
Pre-treatment metastatic workup of the study participants. CT = computed tomography, MRI = magnetic resonance imaging, USG = ultrasonography, CXR = chest X-ray. The modalities for pre-treatment workup undertaken by the patients were not mutually exclusive.

All patients underwent metastatic workup before the commencement of treatment. The most widely used imaging study was tecnitium-99 bone scintigraphy (bone scan) (90.0%) followed by chest radiography (chest X-ray) (76.2%) and abdominopelvic ultrasonography (73.0%). Magnetic resonance imaging (9.8%) and CT scan (4.1%) were also used albeit by relatively few patients (Figure 1). None of the patients had a positron emission tomography scan done mainly due to its unavailability within the country. Each patient underwent at least two different imaging investigations. No metastatic lesions were detected in any of the imaging studies for all patients.

**Figure 2.**
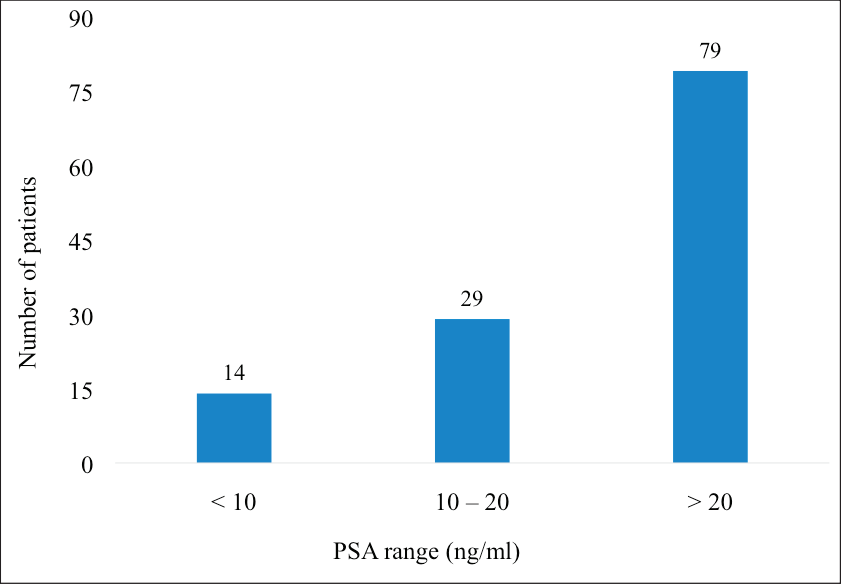
iPSA of patients with localised CaP. PSA = prostate specific antigen, CaP = prostate cancer. For patients with multiple PSA results of different values done prior to starting treatment, the iPSA was taken to be the highest pretreatment PSA value of the patients.

The majority of the patients (*n* = 79, 64.8%) had iPSA >20 ng/mL (high-risk patients) whereas only 14 (11.5%) had iPSA values <10 ng/mL. The lowest iPSA recorded among the patients was 3.98 ng/mL whereas the highest value was 705 ng/mL (Figure 2).

**Figure 3.**
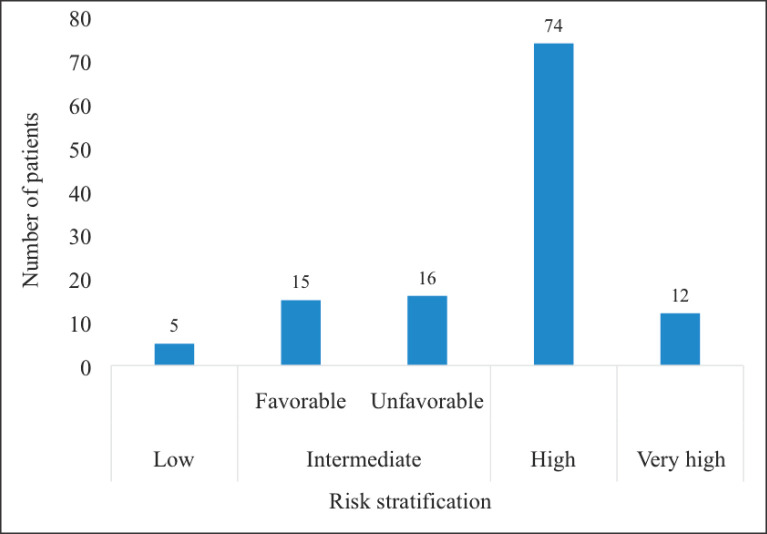
Risk stratification of patients with localised CaP. CaP = Prostate cancer. The intermediate risk group was divided into the favourable and unfavourable intermediate risk groups. There were no patients in the category of very low risk CaP.

Majority (*n* = 74, 60.7%) of the patients had high risk localised CaP whereas only 5 (4.1%) had low risk disease: There were no patients with very low risk CaP (Figure 3).

*Comorbidities*

**Figure 4.**
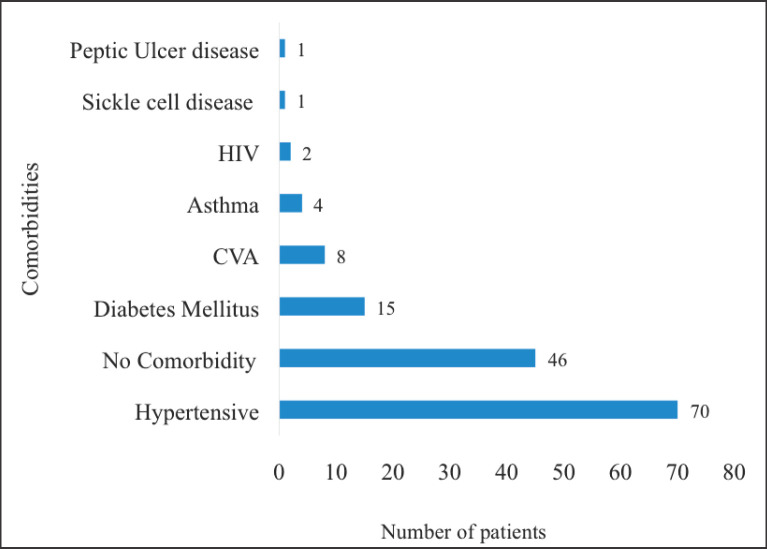
Comorbidities of patients with localised CaP. HIV: human immunodeficiency virus, CVA: cardiovascular accident. Hypertensives were patients with known hypertensive heart disease whereas ‘asthma’ signifies patients with bronchial asthma. These comorbidities were not mutually exclusive. There were some patients who had more than one of the listed medical conditions simultaneously.

Hypertensive heart disease (*n* = 70, 57.4%) was the most prevalent comorbidity among the participants of the study followed by DM (*n* = 15, 12.3%), cardiovascular accidents (*n* = 8, 6.5%) and bronchial asthma (*n* = 4, 3.3%). On the other hand, 46 (37.7%) participants did not have any comorbidities at all (Figure 4).

*Clinical symptoms*

**Figure 5.**
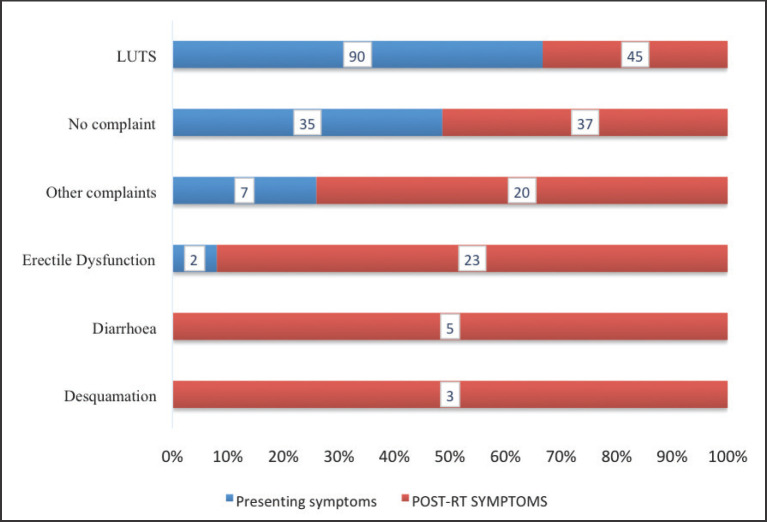
Patients’ symptoms pre and post EBRT. LUTS = lower urinary tract symptoms, EBRT = external beam radiotherapy. Presenting symptoms were the complaints experienced and expressed by the patients at the time of their presentation at the hospital before receiving treatment. ‘No complaint’ refers to patients who did not present with or complain of any symptoms before starting their treatment.

Comparison of patients’ symptoms prior to and post-EBRT is presented in Figure 5. Thirty-five patients had no symptoms prior to receiving treatment compared to 37 who were asymptomatic within 3 months of completing their treatment. There was a decrease in the number of patients with lower urinary tract symptoms from 90 (73.8%) to 45 (36.9%). There were no patients who had either diarrhaea or desquamation prior to treatment with EBRT, however these complaints were recorded among 5 (4.1%) and 5 (2.5%) patients, respectively, after treatment. There was an increase of erectile dysfunction from 2 (1.6%) to 23 (18.9%).

*Androgen deprivation therapy*

**Figure 6.**
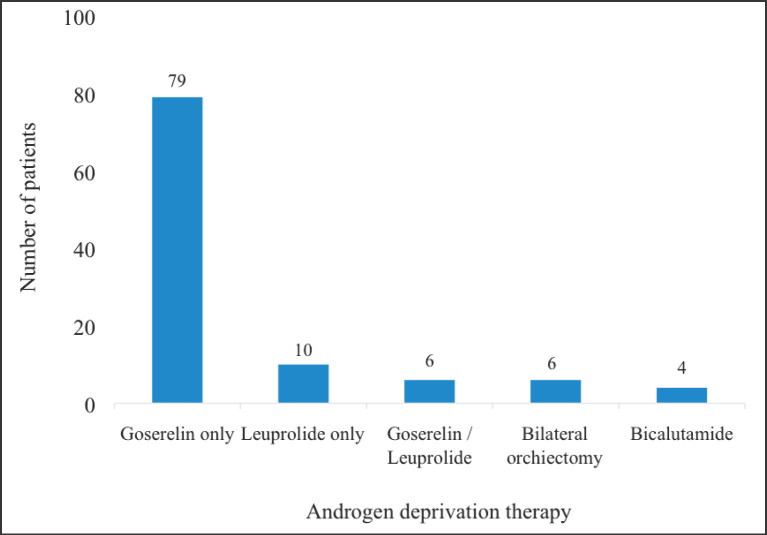
ADT received by patients. The figure summarises the hormonal ADT received by the patients. Some received alternate injections of Goserelin (Zoladex, 11.8 mg) and leuprolide 11.25 mg. This was either due to the unavailability of one of the drugs at some point during treatment or due to financial considerations. The cost of Goserelin was about twice that of leuprolide so patients receiving the former who could no longer afford it often switched to the latter. These medications were administered as 3-monthly injections. Some patients also received Bicalutamide (Casodex 50 mg/150 mg).

Majority of the patients who received ADT (*n* = 79) were put on 3-monthly injections of 10.8 mg of Goserelin (analogue of Gonadotropin Releasing Hormone – GnRH). Ten patients also received Leuprolide whereas four patients received Bicalutamide, an androgen receptor blocker (Figure 6).

**Figure 7.**
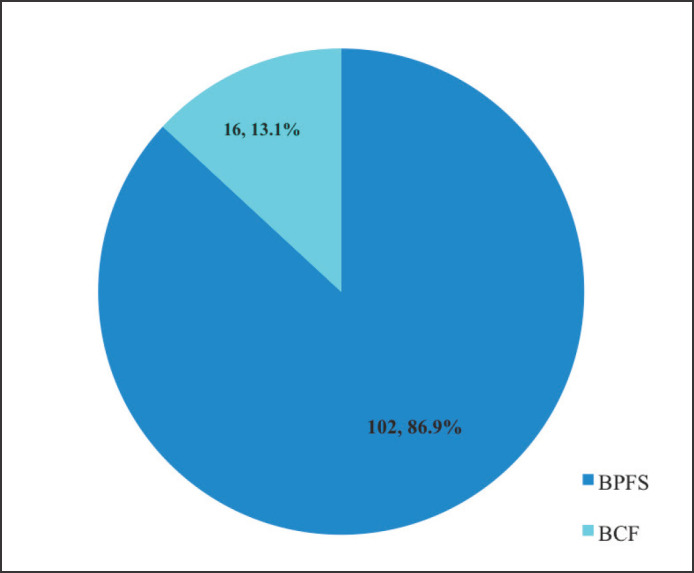
Biochemical outcome after EBRT in localised CaP. BPFS = biochemical progression-free survival, BCF = biochemical failure, EBRT = external beam radiotherapy.

The majority (*n* = 106, 86.9%) of the patients were free of biochemical progression whereas 16 (13.1%) experienced BCF after a mean follow up period of 31.3 months (Figure 7).

**Figure 8.**
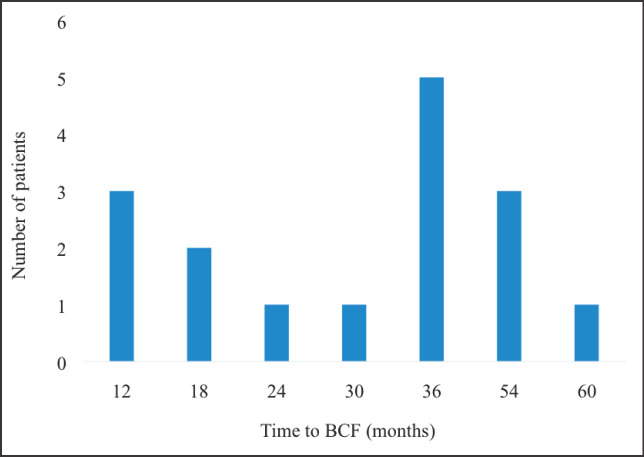
Pattern of time to BCF. BCF – biochemical failure.

Figure 8 displays the time to BCF among patients treated with EBRT for localised CaP. The shortest time to BCF was 12 months whereas the longest was 60 months. Majority of the patients who experienced BCF did so at 36 months post curative treatment with EBRT.

Table 2 illustrates the relationship between the clinicopathological characteristics of the patients and biochemical outcomes. The results demonstrate a positive statistically significant correlation between BCF and percentage of cores positive, grade group and risk stratification. Patients with ≥50% cores positive were more likely to experience BCF than those with <50% cores positive (CI: 0.012 – 0.136, *p* = 0.025). The higher the risk stratification above the favorable intermediate group, the higher the risk of experiencing BCF. Other variables such as age, iPSA, clinical presentation, T-category, comorbidities, smoking and alcohol intake did not show any significant correlation with BCF.

## Discussion

### Clinicopathological characteristics

The modal age range of incidence of CaP in Ghana is estimated to be from 70 to 79 years with the lowest occurrence recorded among men who are either between 40 and 49 years or more than 100 years old [18]. In this study, however, most of the patients with localised CaP were between 60 and 69 years old. The mean age of patients was 67.8 years (SD ± 6.1), consistent with the results of a previous study among Ghanaian males which reported a mean population age was 66.2 years (SD ±  7.1) among patients with CaP [19]. The findings of this study are not surprising because the vast majority of CaP cases are diagnosed in men above 65 years [20]. A study in Kenya reported a mean age of 67 years with 87.5% of the participants above the age of 61 years and the highest incidence found in the 66–70 years age range [21]. The youngest patient in this study was 53 years old and the oldest was 81 years. CaP incidence increases with increasing age. Previous studies have reported that men under the age of 39 years have a 0.005% risk of developing CaP; those aged 40 to 59 have a 2.2% chance whereas men aged 60 to 79 have a 13.7% chance [22]. CaP is considered a disease of the elderly since men over 65 years account for 75% of all diagnoses whereas the disease remains uncommon among men under the age of 40 years [23, 24]. Early age at diagnosis can be attributed to widespread screening which translates into CaP detection among asymptomatic men. Similarly, asymptomatic men who are screened late will be diagnosed late as well.

All the patients had good performance status of either ECOG 0 or 1. This is essentially because the study involved only patients with localised disease which is associated with less morbidity than advanced disease. It is common for patients with advanced or metastatic CaP to present with poor performance status which can be a reason to withhold aggressive therapy for the management of the disease. Even though 62.3% of the patients had comorbidities, their overall performance status was good, making them more likely to receive full treatment.

Among the patients, 41% had a history of alcohol intake whereas 11.5% were smokers. Smoking is a well-established risk factor for the development of CaP whereas no such relationship has been demonstrated between alcohol intake and CaP. Compared to never smokers, current smoking is reported to confer a 40% increase in CaP risk. The role of smoking in prostate carcinogenesis is related to either exposure to carcinogens contained in cigarettes such as cadmium and/or increased circulatory hormonal levels [25]. Consideration of potential adverse effects is one of the key factors considered by patients when choosing between definitive RT and radical prostatectomy for the management of localised CaP. There is a plethora of misconceptions and myths about the dangers associated with radiation therapy vis-à-vis patient’s quality of life. The results of this study clearly demonstrate improvement of patient’s symptoms with radiation therapy. Prior to treatment, 75% of the patients had lower urinary tract symptoms and after completion of treatment only 37.5% still had these symptoms. This implies that half of the patients saw resolution of their lower urinary tract symptoms with radiation treatment. Also, there was a modest increase in the number of asymptomatic patients from 29% to 30.8% prior and post radiation treatment, respectively. Diarrhaea and desquamation were experienced exclusively after patients had undergone RT. These symptoms were acute side effects associated with CaP irradiation. They can potentially impact negatively on the quality of life of the CaP patient. In our study, only 6.7% of the patients experienced either of these radiation-induced toxicities.

### Biochemical outcome

Out of 122 patients, only 16 (13.1%) experienced BCF at after a mean follow-up of 31.3 months. The percentage of patients who were free from BCF according to risk stratification was 100%, 100%, 93.8%, 83.8% and 75% for low, favorable intermediate, unfavourable intermediate, high-risk and very high-risk groups, respectively. This finding is more favorable than that of a previous study which reported a 5-year biochemical progression-free survival (BPFS) of 62% among patients with localised disease [26]. Another study reported BPFS of 87%, 61% and 28% in the low, intermediate and high-risk groups, respectively, following EBRT [8]. Similar improvement in biochemical outcome was observed by other authors who opined that early detection and therapeutic advancement have both contributed to the observed breakthroughs in CaP control. This explains the notable disease control and survival outcomes observed over the past 20 years. Men with high-risk diseases have had the largest increase in escape from PSA failure even though all patients have benefited. This study's findings show that RT is effective in the management of localised CaP in terms of BPFS regardless of the risk group.

### Factors associated with biochemical outcome

This study demonstrates that the occurrence of BCF cuts across all ages. There was no statistically significant relationship between age at diagnosis and BCF (*p* = 0.417). The reason for this finding could likely be that irrespective of the age of the patients in this study, majority (94.3%) presented with a performance status of ECOG 0 which gave the opportunity for an optimal curative approach such as the use of ADT and escalated-dose RT. This study is also consistent with another study that demonstrated that sociodemographic characteristics do not significantly affect the prognosis of CaP patients who receive EBRT [27]. The results show that 75% of the patients with BCF initially presented with symptoms. The majority of these complaints were unspecified lower urinary tract symptoms. However, there was no statistically significant correlation between either clinical presentation or patients’ comorbidity and biochemical outcome, *p* = 0.729 and *p* = 0.294, respectively.

A significant predictor of the biochemical response of CaP treated with EBRT is the percentage of preoperative biopsy cores positive [28]. A direct relationship between the number of cores positive and biochemical outcome has been described in detail [29]. When discussing PSA outcome after EBRT with newly diagnosed and clinically localised CaP patients, it is important to consider the percentage of prostate biopsy cores that are positive. In this study, 81.3% of cases with BCF had ≥50% cores positive. There was a strong correlation between percentage of cores positive and BCF (*p* = 0.025). This implies that the higher the percentage of cores positive, the higher the likelihood of BCF. This is consistent with results of previous studies. For example, in one study, it was observed that patients had a 5-year biochemical control rate of 83% when their percentage core positivity was less than 33%, but only 57% when percentage core positivity was greater than 67% [28]. The percentage of cores positive has been proposed as a potential predictive factor to improve the current risk classification for CaP patients receiving EBRT [30].

The results of this study show a direct correlation between iPSA and the likelihood of biochemical progression. The higher the iPSA, the higher the likelihood of BCF. iPSA is a key factor considered in risk stratification and treatment decision making for localised CaP [31]. The correlation found in this study however did not rise to the level of statistical significance.

BCF was predominant among patients with T2b, T2c and T3a disease. Total biochemical outcome according to T – Category in this study is: 100%, 93.3%, 96%, 77%, 86.7% and 75% for T1a, T1c, T2a, T2b, T2c and T3a, respectively. This finding is not surprising since it has been established that tumour size and local extent are significant predictors of biochemical recurrence in patients with CaP [32]. The results of this study is consistent with a previous study reporting that at 5 years, patients with stage T1c, T2a, T2b, T2c and T3 had biochemical disease-free rates of 74%, 79%, 72%, 24% and 33%, respectively, revealing BCF to be predominant in T2c and T3 [33].

The Gleason scoring system is a reliable indicator of disease behaviour and the most accurate prognostic factor for predicting clinical outcome for CaP [34]. In this study, the Gleason Score was used to Grade the patients into group 1–5 depending on their Gleason pattern. Cases of BCF were recorded among patients in grade groups 3 and 4. There was a strong correlation between grade group and BCF (*p* = 0.001). This is consistent with another study that demonstrated that Gleason score ≥7 is linked to an increased risk of biochemical recurrence [35].

Majority of the patients (66.4%) received 3-dimensional radiotherapy (3DCRT) on a cobalt-60 teletherapy machine whereas the rest were treated on a 6 MV Linac. In Ghana and other developing countries, patients are still treated with cobalt-60 machines although these have been replaced in the developed world. Considered outmoded according to modern RT delivery standards, the cobalt-60 teletherapy machine remains the workhorse of several RT centers in Africa. It presents patients with the advantage of lower cost of treatment with similar treatment outcomes as the 6 MV linear accelerator. In all, 16% of patients treated on the cobalt-60 teletherapy machine had BCF whereas biochemical progression was recorded among only 7.3% of those who received treatment on the 6 MV Linac. This difference, however, did not rise to the level of statistical significance (*p* = 0.180).

All the patients who experienced biochemical progression in this study had unfavourable intermediate risk disease or higher. There is a direct correlation between BCF and patient’s stratification (*p* = 0.019). The results of this study are similar to the study conducted by *Emam et al* (2021) [36] which had a 3- and 5-year BPFS of 86% and 75%, respectively, following EBRT in high and very high-risk patients. There is no doubt that high and very-high-risk patients are far more prone to BCF than those in lower-risk groups.

## Limitations

This research was a single-facility based cross-sectional study conducted in Ghana. Patients were recruited at the largest of the three RT and oncology treatment centers in the country. However, the BCF rate reported in this study might not necessarily be the same at the other RT sites in the country due to the possibility of inhomogeneous patient profiles. Additionally, the size of the patient population was such that after patient stratification the number of patients in some of the strata appeared to be small for stringent statistical analysis. The evaluation of patients’ symptoms after RT was done at 3 months post treatment. This duration is short and does not allow sufficient time for the evaluation of long-term effects of radiation treatment on either the quality of life or symptoms of the patients.

## Conclusion

There was a low rate of BCF among patients treated with EBRT for localised CaP in Ghana. Biochemical progression was more common among patients treated on the Cobalt-60 teletherapy machine compared to those treated with the 6 MV Linac but this difference was not statistically significant (*p* = 0.180). Patients’ age, comorbidities and clinical presentation were not associated with biochemical outcome. Strong prognostic factors of biochemical outcome demonstrated in this study were the percentage of cores positive, ISUP grade group and risk stratification. RT produced complete resolution of symptoms in some patients. Diarrhaea and desquamation were symptoms of patients exclusively attributable to treatment with EBRT.

## Conflicts of interest

Authors have no conflicts of interests.

## Funding

This work was supported by the International Atomic Energy Agency.

## Data availability

The data used to support the findings of this study are available from the corresponding author upon reasonable request.

## Figures and Tables

**Table 1.** Baseline clinicopathological characteristics of the study participants (*n* = 122).

Characteristics	Variables	Number of patients	Percentage (%)/SD
Age (years)	Mean	67.6	6.2
	<60	14	11.5
	60–69	57	46.7
	70–79	49	40.2
	> 80	2	1.6
Performance status	ECOG 0	115	94.3
	ECOG 1	7	5.7
History of smoking	Yes	14	11.5
	No	88	72.1
	Unspecified	20	16.4
History of alcohol intake	Yes	50	41.0
	No	52	42.6
	Unspecified	20	16.4
Comorbidities	Present	76	62.3
	Absent	46	37.7
Histology	Adenocarcinoma	122	100
Percentage cores positive	<50%	49	40.2
	≥50%	61	50.0
	Unspecified	12	9.8
T – Category	T1a	3	2.5
	T1c	15	12.3
	T2a	25	20.5
	T2b	22	18.0
	T2c	45	36.9
	T3a	12	9.8
ISUP grade group	1	40	32.8
	2	33	27.0
	3	22	18.0
	4	21	17.2
	5	6	5.0
Radiation dose	≤72 Gy	21	17.2
	74 Gy	81	66.4
	76 Gy	8	6.6
	78 Gy	12	9.8
Radiation treatment machine	Cobalt-60	81	66.4
	6 MV Linac	41	33.6

**Table 2.** Factors associated with biochemical outcome.

Variable	Total (*N* = 122)	BCF (*n* = 16)	*p*-value/CI – 95%
Age <60 60–69 70–79 ≥80	14 57 49 2	4 (28.6%) 5 (8.8%) 7 (14.3%) 0 (0%)	*p* – 0.417 CI (−0.124–0.052)
Clinical symptoms Yes No	87 35	12 (13.8%) 4 (11.4%)	*p* – 0.729 CI (−0.158–0.111)
% Cores positive <50 ≥50	49 61	3 (6%) 13 (21.3%)	*p* – 0.025 CI (0.020–0.284)
T – Category T1a T1c T2a T2b T2c T3a	3 15 25 22 45 12	0 (0%) 1 (6.7%) 1 (4%) 5 (22.7%) 6 (13.3%) 3 (25%)	*p* – 0.085 CI (−0.006–0.087)
iPSA >10 10–20 >20	14 29 79	1 (7%) 2 (7%) 13 (16.5%)	*p* – 0.181 CI (−0.028–0.147)
Grade group 1 2 3 4 5	40 33 22 21 6	1 (2.5%) 2 (6%) 6 (27.3%) 5 (23.8%) 2 (33.3%)	*p* – 0.001 CI (0.036–0.131)
Comorbidities Yes No	77 45	12 (15.6%) 4 (9%)	*p* – 0.294 CI (−0.193–0.059)
Risk group Low risk Favourable intermediate Unfavourable intermediate High risk Very high risk	5 15 16 74 12	0 (0%) 0 (0%) 1 (6.2%) 12 (16.2%) 3 (25%)	*p* – 0.019 CI (0.012–0.136)
Smoking Yes No Unspecified	14 88 20	1 (7%) 11 (12.5%) 4 (20%)	*p* – 0.501 CI (−0.053–0.108)
Alcohol Yes No Unspecified	50 52 20	7 (14%) 5 (10%) 4 (20%)	*p* – 0.239 CI (−0.034–0.134)
EBRT dose ≤72 Gy 74 Gy 76 Gy 78 Gy	21 81 8 12	3 (14,3%) 11 (13.6%) 0 (0%) 2 (16.7%)	*p* – 0.882 CI (−0.083–0.071)
Treatment machine Cobalt-60 Linac	81 41	13 (16%) 3 (7.3%)	*p* – 0.180 CI (−0.216–0.041)

BCF = biochemical failure, PSA = prostate specific antigen, EBRT = external beam radiotherapy

## References

[1] Sung H, Ferlay J, Siegel RL (2021). Global cancer statistics 2020: GLOBOCAN estimates of incidence and mortality Worldwide for 36 cancers in 185 countries. CA Cancer J Clin.

[2] Amoako YA, Awuah B, Larsen-Reindorf R (2019). Malignant tumours in urban Ghana: evidence from the city of Kumasi. BMC Cancer.

[3] Necku JG, Anaba EA, Abuosi AA (2019). Prostate cancer awareness and attitude toward early detection among male soldiers in Ghana: a cross-sectional study. Afr J Urol.

[4] Adeloye D, David RA, Aderemi AV (2016). An estimate of the incidence of prostate cancer in Africa: a systematic review and meta-analysis. PLoS One.

[5] Chu LW, Ritchey J, Devesa SS (2011). Prostate cancer incidence rates in Africa. Prostate Cancer.

[6] Dimakakos A, Armakolas A, Koutsilieris M (2014). Novel tools for prostate cancer prognosis, diagnosis, and follow-up. Biomed Res Int.

[7] Lojanapiwat B, Anutrakulchai W, Chongruksut W (2014). Correlation and diagnostic performance of the prostate-specific antigen level with the diagnosis, aggressiveness, and bone metastasis of prostate cancer in clinical practice. Prostate Int.

[8] D'Amico AV, Schultz D, Loffredo M (2000). Biochemical outcome following external beam radiation therapy with or without androgen suppression therapy for clinically localized prostate cancer. JAMA.

[9] Zumsteg ZS, Spratt DE, Romesser PB (2015). The natural history and predictors of outcome following biochemical relapse in the dose escalation era for prostate cancer patients undergoing definitive external beam radiotherapy. Eur Urol.

[10] Kupelian PA, Elshaikh M, Reddy CA (2002). Comparison of the efficacy of local therapies for localized prostate cancer in the prostate-specific antigen era: a large single-institution experience with radical prostatectomy and external-beam radiotherapy. J Clin Oncol.

[11] Yeboah ED, Hsing AW, Mante S (2016). Management of prostate cancer in Accra, Ghana. J West Afr Coll Surg.

[12] Morris WJ, Pickles T, Keyes T (2018). Using a surgical prostate-specific antigen threshold of> 0.2 ng/mL to define biochemical failure for intermediate-and high-risk prostate cancer patients treated with definitive radiation therapy in the ASCENDE-RT randomized control trial. Brachytherapy.

[13] Roach III M, Hanks G, Thames H (2006). Defining biochemical failure following radiotherapy with or without hormonal therapy in men with clinically localized prostate cancer: recommendations of the RTOG-ASTRO Phoenix Consensus Conference. Int J Radiat Oncol* Biol* Phys.

[14] Boorjian SA, Thompson RH, Tollefson MK (2011). Long-term risk of clinical progression after biochemical recurrence following radical prostatectomy: the impact of time from surgery to recurrence. Eur Urol.

[15] Pound CR, Partin AW, Eisenberger MA (1999). Natural history of progression after PSA elevation following radical prostatectomy. JAMA.

[16] Zagars GK, Pollack A (1997). Kinetics of serum prostate-specific antigen after external beam radiation for clinically localized prostate cancer. Radiother Oncol.

[17] Rosario E, Rosario DJ (2020). Localized Prostate Cancer.

[18] Egote AK, Ossei PP, Agyeman-Duah E (2019). Patterns and presentation of prostate cancer in the Brong Ahafo Region of Ghana: a 6-year single center retrospective study. Health.

[19] Calys-Tagoe BN, Yarney J, Kenu E (2014). Profile of cancer patients’ seen at Korle Bu teaching hospital in Ghana (A cancer registry review). BMC Res Notes.

[20] Hussein S, Satturwar S, Van der Kwast T (2015). Young-age prostate cancer. J Clin Pathol.

[21] Wasike R, Magoha G (2007). Descriptive case series of patients presenting with cancer of the prostate and their management at Kenyatta National Hospital, Nairobi. East Afr Med J.

[22] Ng KL (2021). The Etiology of Prostate Cancer.

[23] Rawla P (2019). Epidemiology of prostate cancer. World J Oncol.

[24] Dunn MW, Kazer MW (2011). Prostate Cancer Overview. Semin Oncol Nurs.

[25] Yeargan R, Maiti IB, Nielsen MT (1992). Tissue partitioning of cadmium in transgenic tobacco seedlings and field grown plants expressing the mouse metallothionein I gene. Transgenic Res.

[26] Rosser CJ, Kuban DA, Lee SJ (2004). Racial influence on biochemical disease-free survival in men treated with external-beam radiotherapy for localized prostate cancer. J Natl Med Assoc.

[27] Movsas A, Ibrahim R, Elshaikh MA (2016). Do sociodemographic factors influence outcome in prostate cancer patients treated with external beam radiation therapy?. Am J Clin Oncol.

[28] Kestin LL, Goldstein NS, Vicini FA (2002). Percentage of positive biopsy cores as predictor of clinical outcome in prostate cancer treated with radiotherapy. J Urol.

[29] D'Amico AV, Schultz D, Silver B (2001). The clinical utility of the percent of positive prostate biopsies in predicting biochemical outcome following external-beam radiation therapy for patients with clinically localized prostate cancer. Int J Radiat Oncol Biol Phys.

[30] Huang J, Vicini FA, Williams SG (2012). Percentage of positive biopsy cores: a better risk stratification model for prostate cancer?. Int J Radiat Oncol Biol Phys.

[31] Rodrigues G, Warde P, Pickles T (2012). Pre-treatment risk stratification of prostate cancer patients: a critical review. Can Urol Assoc J.

[32] Eichelberger LE, Koch MO, Eble JN (2005). Maximum tumor diameter is an independent predictor of prostate-specific antigen recurrence in prostate cancer. Mod Pathol.

[33] Uchida T, Shoji S, Nakano M (2009). Transrectal high-intensity focused ultrasound for the treatment of localized prostate cancer: eight-year experience. Int J Urol.

[34] Pessoa R, Werahera PN, Kim FJ, Harken AH, Moore EE (2018). Prostate cancer. Abernathy's Surgical Secrets.

[35] Epstein JI (2002). Pathology of prostatic neoplasia. Campbell’s Urol.

[36] Emam A, Hermann G, Attwood K (2021). Oncologic outcome of radical prostatectomy versus radiotherapy as primary treatment for high and very high risk localized prostate cancer. The Prostate.

